# Bis{*N*-[(diethyl­amino)­dimethyl­sil­yl]anilinido-κ^2^ *N*,*N*′}nickel(II)

**DOI:** 10.1107/S1600536812008550

**Published:** 2012-03-03

**Authors:** Juan Chen, Jing Li

**Affiliations:** a Department of Chemistry, Taiyuan Teachers College, Taiyuan 030031, People’s Republic of China; b College of Chemistry and Chemical Engineering, Shanxi University, Taiyuan, 030006, People’s Republic of China

## Abstract

The mononuclear Ni^II^ amide, [Ni(C_12_H_21_N_2_Si)_2_], has the Ni^II^ atom *N*,*N*′-chelated by the *N*-silylated anilinide ligands. The ligands are arranged *cis* to each other and obey the *C*_2_-symmetry operation. The two ends of the N—Si—N chelating unit exhibit different affinities for the metal atom: the Ni—N_anilinide_ bond length is 1.913 (3) Å and Ni—N_amine_ is 2.187 (3) Å. The four-coordinate Ni^II^ ion demonstrates a distorted tetra­hedral geometry.

## Related literature

For related reviews of metal amides, see: Holm *et al.* (1996 ▶); Kempe (2000 ▶). For the catalytic applications of related *N*-silylated anilinide group 4 metal compounds towards olefin polymerization, see: Gibson *et al.* (1998 ▶); Hill & Hitchcock (2002 ▶); Yuan *et al.* (2010 ▶); Zai *et al.* (2010 ▶). For related organometallic compounds with analogous anilinide ligands, see: Schumann *et al.* (2000 ▶); Chen (2008 ▶, 2009 ▶).
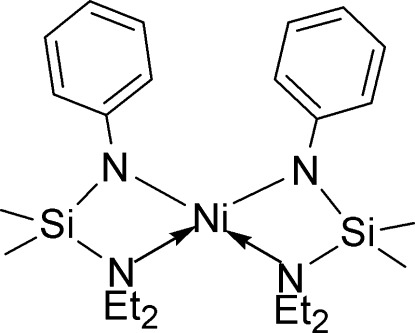


## Experimental

### 

#### Crystal data

[Ni(C_12_H_21_N_2_Si)_2_]
*M* *_r_* = 501.49
Orthorhombic, 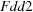

*a* = 21.2631 (11) Å
*b* = 30.0347 (16) Å
*c* = 8.6228 (5) Å
*V* = 5506.8 (5) Å^3^
*Z* = 8
Mo *K*α radiation
μ = 0.81 mm^−1^
*T* = 295 K
0.25 × 0.20 × 0.20 mm

#### Data collection

Bruker SMART CCD diffractometer
Absorption correction: multi-scan (*SADABS*; Sheldrick, 1996 ▶) *T*_min_ = 0.823, *T*_max_ = 0.855
6217 measured reflections
2414 independent reflections
2145 reflections with *I* > 2σ(*I*)
*R*_int_ = 0.029

#### Refinement

*R*[*F* ^2^ > 2σ(*F* ^2^)] = 0.040
*wR*(*F* ^2^) = 0.104
*S* = 1.04
2414 reflections
141 parameters
1 restraint
H-atom parameters constrained
Δρ_max_ = 0.48 e Å^−3^
Δρ_min_ = −0.20 e Å^−3^
Absolute structure: Flack (1983 ▶), 1038 Friedel pairs
Flack parameter: 0.012 (17)

### 

Data collection: *SMART* (Bruker, 2000 ▶); cell refinement: *SAINT* (Bruker, 2000 ▶); data reduction: *SAINT*; program(s) used to solve structure: *SHELXS97* (Sheldrick, 2008 ▶); program(s) used to refine structure: *SHELXL97* (Sheldrick, 2008 ▶); molecular graphics: *SHELXTL* (Sheldrick, 2008 ▶); software used to prepare material for publication: *SHELXL97*.

## Supplementary Material

Crystal structure: contains datablock(s) I, global. DOI: 10.1107/S1600536812008550/rk2340sup1.cif

Structure factors: contains datablock(s) I. DOI: 10.1107/S1600536812008550/rk2340Isup2.hkl

Additional supplementary materials:  crystallographic information; 3D view; checkCIF report

## Figures and Tables

**Fig. 1.**
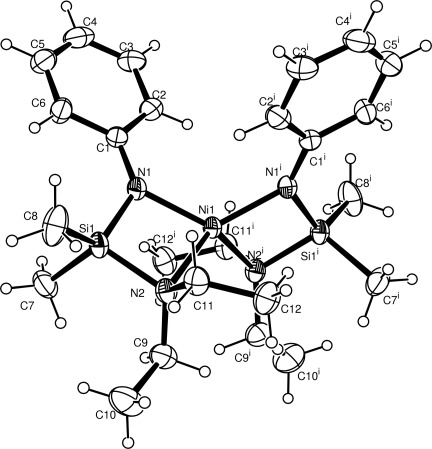
The molecular structure, showing the atom–numbering scheme. Displacement ellipsoids are drawn at the 30% probability level. H atoms are presented as a small spheres of arbitrary radius. Symmetry codes: (i) -x+3/2, -y+1/2, z.

## supplementary crystallographic information

### Comment

Metal amides were important substitutes for cyclopentadienyl derivatives and were found to have valuable applications in various industrial and biological processes (Holm *et al.*, 1996; Kempe, 2000). Group 4 metal amides with the *N*-silylated anilinide ligands were active catalysts for olefin polymerization (Gibson *et al.*, 1998; Hill & Hitchcock, 2002). Our research interest focused on *N*-silylated anilinide ligands bearing a pendant amino group. Analogous compounds with different metals including Zn (Schumann *et al.*, 2000), Zr (Chen, 2009) and Fe (Chen, 2008) have been synthesized and the zirconium compounds were reported showing good performance in ethylene polymerization (Yuan *et al.*, 2010). Recently, a kind of bidentate *N*–donor ligand supported nickel complex activated by MAO was used as a catalyst conducting longstanding living ethylene polymerization (Zai *et al.*, 2010). In view of the importance of these compounds, the synthesis and crystal structure of a new nickel(II) anilinide complex is reported.

The title compound was prepared by one–pot reaction of Li*Bu^n^*, *N*–[(diethylamino)dimethylsilyl]aniline and NiCl_2_. It is monomeric and the ligand has an N—Si—N chelating group. It is presumed that the empty *d*–orbitals on silicon would interact with the lone–pair electrons on the *p*–orbital of nitrogen center through a *d···pπ* interaction, resulting in a "quasi" conjugated N—Si—N motif. Compared with rigid N—C—N chelating unit in the amidinate ligand, the N1—Si1—N2 chelating group is much flexible. The Ni center is fixed by two ligands. Each ligand bites the center with an N1—Ni1—N2 angle of 77.82 (11)°. As biting the metal center, the angle of N1—Si1—N2 is constrained to be 95.28 (13)°. The two ends of the N—Si—N chelating unit exhibit different affinities for the metal center. Ni—N_anilinide_ bond is 1.913 (3) Å and Ni—N_amino_ bond is 2.187 (3)Å. The coordinate geometry of N_anilinide_ atom is trigonal planar (sum of three angles around it being 359°). Both distances of Si1—N1 (1.699 (3)Å) and N1—C1 (1.375 (4)Å) are short. It suggests a certain degree delocalization of the lone–pair electron density from the *p*–orbital of N1 to the *π*–orbital of the phenylsubstituent. The two ligands around the Ni atom are arranged *cis* to each other and obey the *C*_2_ symmetry operation. The four-coordinate Ni atom demonstrates a distorted tetrahedral geometry.

### Experimental

A solution of Li*Bu^n^* (1.6 *M*, 1.9 ml, 3.0 mmol) in hexane was slowly added into a solution of *N*–[(diethylamino)dimethylsilyl]aniline (0.67 g, 3.0 mmol) in *THF* (20 ml) at 273 K by syringe. The mixture was stirred at room temperature for two hours and then added to a stirring suspension of NiCl_2_ (0.20 g, 1.5 mmol) in *THF* (20 ml) at 273 K. The resulting mixture was stirred at room temperature for 8 h. Then all the volatiles were removed under vacuum. The residue was extracted with toluene (25 ml). The filtrate was concentrated to give the title compound as red crystals (yield 0.39 g, 52%). M.p.: 451–452 K. MS (EI, 70 eV): *m/z* 502 [*M*]^+^. Anal. Calc. for C_24_H_42_Ni_2_N_4_Si_2_: C, 57.48; H, 8.44; N, 11.17%. Found: C, 56.99; H, 8.13; N, 10.93%.

### Refinement

The methyl H atoms were constrained to an ideal geometry, with C—H distances of 0.96Å and *U*_iso_(H) = 1.5*U*_eq_(C), but each group was allowed to rotate freely about its C–C and C–Si bonds. The methylene H atoms were constrained with C—H distances of 0.97Å and *U*_iso_(H) = 1.2*U*_eq_(C). The phenyl H atoms were placed in geometrically idealized positions and constrained to ride on their parent atoms, with C—H distances in the range 0.93Å and *U*_iso_(H) = 1.2*U*_eq_(C).

### Crystal data

**Table:**

[Ni(C_12_H_21_N_2_Si)_2_]	*D*_x_ = 1.210 Mg m^−^^3^
*M**_r_* = 501.49	Melting point = 451–452 K
Orthorhombic, *F**d**d*2	Mo *K*α radiation, λ = 0.71073 Å
Hall symbol: F 2 -2d	Cell parameters from 2035 reflections
*a* = 21.2631 (11) Å	θ = 2.1–26.4°
*b* = 30.0347 (16) Å	µ = 0.81 mm^−^^1^
*c* = 8.6228 (5) Å	*T* = 295 K
*V* = 5506.8 (5) Å^3^	Block, red
*Z* = 8	0.25 × 0.20 × 0.20 mm
*F*(000) = 2160	

### Data collection

**Table:**

Bruker SMART CCD diffractometer	2414 independent reflections
Radiation source: fine-focus sealed tube	2145 reflections with *I* > 2σ(*I*)
Graphite monochromator	*R*_int_ = 0.029
φ and ω scan	θ_max_ = 25.5°, θ_min_ = 2.4°
Absorption correction: multi-scan (*SADABS*; Sheldrick, 1996)	*h* = −25→22
*T*_min_ = 0.823, *T*_max_ = 0.855	*k* = −36→34
6217 measured reflections	*l* = −10→10

### Refinement

**Table:**

Refinement on *F*^2^	Secondary atom site location: difference Fourier map
Least-squares matrix: full	Hydrogen site location: inferred from neighbouring sites
*R*[*F*^2^ > 2σ(*F*^2^)] = 0.040	H-atom parameters constrained
*wR*(*F*^2^) = 0.104	*w* = 1/[σ^2^(*F*_o_^2^) + (0.0724*P*)^2^] where *P* = (*F*_o_^2^ + 2*F*_c_^2^)/3
*S* = 1.04	(Δ/σ)_max_ < 0.001
2414 reflections	Δρ_max_ = 0.48 e Å^−^^3^
141 parameters	Δρ_min_ = −0.20 e Å^−^^3^
1 restraint	Absolute structure: Flack (1983), 1038 Friedel pairs
Primary atom site location: structure-invariant direct methods	Flack parameter: 0.012 (17)

### Special details

**Table:**

Geometry. All s.u.'s (except the s.u. in the dihedral angle between two l.s. planes) are estimated using the full covariance matrix. The cell s.u.'s are taken into account individually in the estimation of s.u.'s in distances, angles and torsion angles; correlations between s.u.'s in cell parameters are only used when they are defined by crystal symmetry. An approximate (isotropic) treatment of cells.u.'s is used for estimating s.u.'s involving l.s. planes.
Refinement. Refinement of *F*^2^ against ALL reflections. The weighted *R*–factor *wR* and goodness of fit *S* are based on *F*^2^, conventional *R*–factors *R* are based on *F*, with *F* set to zero for negative *F*^2^. The threshold expression of *F*^2^ > σ(*F*^2^) is used only for calculating *R*–factors(gt) *etc*. and is not relevant to the choice of reflections for refinement. *R*–factors based on *F*^2^ are statistically about twice as large as those based on *F*, and *R*–factors based on ALL data will be even larger.

### Fractional atomic coordinates and isotropic or equivalent isotropic displacement parameters (Å^2^)

**Table:**

	*x*	*y*	*z*	*U*_iso_*/*U*_eq_	
Ni1	0.7500	0.2500	0.59583 (5)	0.03740 (17)	
Si1	0.62152 (4)	0.24725 (3)	0.62421 (11)	0.0448 (3)	
N1	0.67563 (11)	0.23000 (10)	0.4923 (4)	0.0430 (6)	
N2	0.67555 (11)	0.28051 (8)	0.7349 (4)	0.0426 (6)	
C1	0.67006 (15)	0.20291 (10)	0.3645 (4)	0.0431 (7)	
C2	0.72350 (19)	0.18285 (13)	0.2987 (4)	0.0564 (9)	
H2A	0.7629	0.1889	0.3405	0.068*	
C3	0.7188 (2)	0.15423 (13)	0.1727 (4)	0.0622 (10)	
H3A	0.7550	0.1413	0.1318	0.075*	
C4	0.6615 (2)	0.14486 (13)	0.1082 (5)	0.0700 (11)	
H4A	0.6585	0.1255	0.0244	0.084*	
C5	0.6092 (2)	0.16427 (14)	0.1681 (5)	0.0663 (11)	
H5A	0.5702	0.1585	0.1233	0.080*	
C6	0.61285 (17)	0.19221 (13)	0.2936 (4)	0.0567 (9)	
H6A	0.5760	0.2045	0.3330	0.068*	
C7	0.58850 (18)	0.20036 (14)	0.7411 (5)	0.0668 (11)	
H7A	0.5435	0.2026	0.7434	0.100*	
H7B	0.6046	0.2019	0.8449	0.100*	
H7C	0.6005	0.1725	0.6949	0.100*	
C8	0.55404 (18)	0.28164 (14)	0.5536 (7)	0.0819 (15)	
H8A	0.5153	0.2690	0.5902	0.123*	
H8B	0.5540	0.2821	0.4423	0.123*	
H8C	0.5582	0.3115	0.5921	0.123*	
C9	0.6760 (2)	0.27464 (13)	0.9052 (5)	0.0579 (9)	
H9A	0.7149	0.2870	0.9452	0.069*	
H9B	0.6764	0.2430	0.9274	0.069*	
C10	0.6211 (2)	0.29574 (18)	0.9940 (7)	0.0892 (15)	
H10A	0.6261	0.2903	1.1030	0.134*	
H10B	0.5822	0.2829	0.9592	0.134*	
H10C	0.6205	0.3273	0.9754	0.134*	
C11	0.67674 (16)	0.32842 (11)	0.6879 (5)	0.0531 (9)	
H11A	0.6419	0.3436	0.7375	0.064*	
H11B	0.6704	0.3303	0.5766	0.064*	
C12	0.73718 (17)	0.35238 (12)	0.7289 (6)	0.0634 (10)	
H12A	0.7347	0.3828	0.6948	0.095*	
H12B	0.7719	0.3379	0.6787	0.095*	
H12C	0.7432	0.3516	0.8392	0.095*	

### Atomic displacement parameters (Å^2^)

**Table:**

	*U*^11^	*U*^22^	*U*^33^	*U*^12^	*U*^13^	*U*^23^
Ni1	0.0262 (3)	0.0458 (3)	0.0402 (3)	−0.0003 (2)	0.000	0.000
Si1	0.0273 (4)	0.0509 (5)	0.0561 (8)	0.0002 (3)	0.0019 (4)	−0.0001 (4)
N1	0.0295 (13)	0.0548 (16)	0.0446 (15)	−0.0033 (11)	−0.0011 (12)	0.0014 (13)
N2	0.0320 (13)	0.0449 (15)	0.0510 (16)	0.0044 (10)	0.0024 (13)	0.0001 (13)
C1	0.0439 (17)	0.0488 (17)	0.0365 (18)	−0.0049 (13)	−0.0020 (14)	0.0065 (14)
C2	0.0478 (19)	0.075 (2)	0.046 (2)	−0.0021 (17)	−0.0036 (16)	−0.0034 (17)
C3	0.073 (3)	0.070 (2)	0.0439 (19)	0.008 (2)	0.004 (2)	−0.0073 (18)
C4	0.106 (3)	0.063 (2)	0.0407 (19)	−0.013 (2)	−0.009 (2)	−0.0014 (18)
C5	0.073 (3)	0.075 (3)	0.052 (2)	−0.018 (2)	−0.012 (2)	0.0021 (19)
C6	0.048 (2)	0.066 (2)	0.056 (2)	−0.0136 (16)	−0.0102 (18)	0.0067 (17)
C7	0.062 (2)	0.070 (2)	0.068 (3)	−0.0159 (19)	0.021 (2)	0.001 (2)
C8	0.0393 (19)	0.080 (3)	0.126 (5)	0.0105 (18)	−0.028 (2)	0.004 (3)
C9	0.063 (2)	0.059 (2)	0.052 (2)	0.0005 (17)	0.0074 (18)	−0.0018 (18)
C10	0.089 (3)	0.102 (3)	0.077 (3)	0.010 (2)	0.027 (3)	−0.012 (3)
C11	0.0448 (18)	0.0486 (19)	0.066 (2)	0.0059 (14)	0.0031 (16)	−0.0046 (16)
C12	0.061 (2)	0.047 (2)	0.082 (3)	−0.0021 (16)	−0.010 (2)	−0.0029 (19)

### Geometric parameters (Å, º)

**Table:**

Ni1—N1	1.913 (3)	C5—C6	1.372 (6)
Ni1—N1^i^	1.913 (3)	C5—H5A	0.9300
Ni1—N2^i^	2.187 (3)	C6—H6A	0.9300
Ni1—N2	2.187 (3)	C7—H7A	0.9600
Ni1—Si1^i^	2.7441 (8)	C7—H7B	0.9600
Ni1—Si1	2.7441 (8)	C7—H7C	0.9600
Si1—N1	1.699 (3)	C8—H8A	0.9600
Si1—N2	1.797 (3)	C8—H8B	0.9600
Si1—C7	1.869 (4)	C8—H8C	0.9600
Si1—C8	1.870 (4)	C9—C10	1.534 (6)
N1—C1	1.375 (4)	C9—H9A	0.9700
N2—C9	1.479 (5)	C9—H9B	0.9700
N2—C11	1.495 (4)	C10—H10A	0.9600
C1—C6	1.399 (5)	C10—H10B	0.9600
C1—C2	1.406 (5)	C10—H10C	0.9600
C2—C3	1.389 (5)	C11—C12	1.515 (5)
C2—H2A	0.9300	C11—H11A	0.9700
C3—C4	1.369 (6)	C11—H11B	0.9700
C3—H3A	0.9300	C12—H12A	0.9600
C4—C5	1.359 (6)	C12—H12B	0.9600
C4—H4A	0.9300	C12—H12C	0.9600
			
N1—Ni1—N1^i^	124.35 (18)	C3—C4—H4A	120.4
N1—Ni1—N2^i^	136.28 (10)	C4—C5—C6	121.0 (4)
N1^i^—Ni1—N2^i^	77.82 (11)	C4—C5—H5A	119.5
N1—Ni1—N2	77.82 (11)	C6—C5—H5A	119.5
N1^i^—Ni1—N2	136.28 (10)	C5—C6—C1	122.3 (4)
N2^i^—Ni1—N2	113.51 (15)	C5—C6—H6A	118.8
N1—Ni1—Si1^i^	150.95 (9)	C1—C6—H6A	118.8
N1^i^—Ni1—Si1^i^	37.73 (9)	Si1—C7—H7A	109.5
N2^i^—Ni1—Si1^i^	40.81 (7)	Si1—C7—H7B	109.5
N2—Ni1—Si1^i^	131.23 (8)	H7A—C7—H7B	109.5
N1—Ni1—Si1	37.73 (9)	Si1—C7—H7C	109.5
N1^i^—Ni1—Si1	150.95 (9)	H7A—C7—H7C	109.5
N2^i^—Ni1—Si1	131.23 (8)	H7B—C7—H7C	109.5
N2—Ni1—Si1	40.81 (7)	Si1—C8—H8A	109.5
Si1^i^—Ni1—Si1	169.77 (4)	Si1—C8—H8B	109.5
N1—Si1—N2	95.28 (13)	H8A—C8—H8B	109.5
N1—Si1—C7	112.69 (18)	Si1—C8—H8C	109.5
N2—Si1—C7	111.89 (18)	H8A—C8—H8C	109.5
N1—Si1—C8	118.0 (2)	H8B—C8—H8C	109.5
N2—Si1—C8	110.88 (17)	N2—C9—C10	116.2 (4)
C7—Si1—C8	107.7 (2)	N2—C9—H9A	108.2
C7—Si1—Ni1	116.41 (13)	C10—C9—H9A	108.2
C8—Si1—Ni1	135.91 (16)	N2—C9—H9B	108.2
C1—N1—Si1	131.2 (2)	C10—C9—H9B	108.2
C1—N1—Ni1	129.1 (2)	H9A—C9—H9B	107.4
Si1—N1—Ni1	98.73 (15)	C9—C10—H10A	109.5
C9—N2—C11	112.6 (3)	C9—C10—H10B	109.5
C9—N2—Si1	117.7 (2)	H10A—C10—H10B	109.5
C11—N2—Si1	113.7 (2)	C9—C10—H10C	109.5
C9—N2—Ni1	119.3 (2)	H10A—C10—H10C	109.5
C11—N2—Ni1	104.02 (19)	H10B—C10—H10C	109.5
Si1—N2—Ni1	86.48 (12)	N2—C11—C12	114.1 (3)
N1—C1—C6	124.1 (3)	N2—C11—H11A	108.7
N1—C1—C2	120.5 (3)	C12—C11—H11A	108.7
C6—C1—C2	115.4 (3)	N2—C11—H11B	108.7
C3—C2—C1	121.5 (4)	C12—C11—H11B	108.7
C3—C2—H2A	119.2	H11A—C11—H11B	107.6
C1—C2—H2A	119.2	C11—C12—H12A	109.5
C4—C3—C2	120.6 (4)	C11—C12—H12B	109.5
C4—C3—H3A	119.7	H12A—C12—H12B	109.5
C2—C3—H3A	119.7	C11—C12—H12C	109.5
C5—C4—C3	119.1 (4)	H12A—C12—H12C	109.5
C5—C4—H4A	120.4	H12B—C12—H12C	109.5

Symmetry code: (i) −*x*+3/2, −*y*+1/2, *z*.
